# Genomic Insights into *Staphylococcus aureus* Isolates Exhibiting Diminished Daptomycin Susceptibility

**DOI:** 10.3390/pathogens13030206

**Published:** 2024-02-26

**Authors:** Natalia Gómez-Casanova, Mª Nieves Gutiérrez-Zufiaurre, Ana Mª Blázquez de Castro, Juan Luis Muñoz-Bellido

**Affiliations:** 1 Research Group on Clinical Microbiology and Parasitology and Antimicrobial Resistance (IIMD-16), Instituto de Investigación Biomédica de Salamanca (IBSAL), Universidad de Salamanca, CSIC, Hospital Universitario de Salamanca, 37007 Salamanca, Spain; mzufoaurre@saludcastillayleon.es (M.N.G.-Z.); anablazcas@yahoo.es (A.M.B.d.C.); jlmubel@usal.es (J.L.M.-B.); 2 Department of Biomedical and Diagnostic Sciences, Universidad de Salamanca, 37007 Salamanca, Spain; 3 Department of Microbiology and Parasitology, Hospital Universitario de Salamanca, 37007 Salamanca, Spain

**Keywords:** daptomycin, mutations, resistance, *Staphylococcus aureus*

## Abstract

Daptomycin is one of the last therapeutic resources for multidrug-resistant gram-positive bacteria. Despite its structural similarities with glycopeptides, its mechanisms of action and resistance are different and in some respects are not completely understood. Mutations in several genes have been associated with daptomycin resistance, especially in *mprF*, *walkR*, *rpoB* and *rpoC*, but their role and importance remain to be elucidated. We have studied mutations in 11 genes, which have been previously associated with daptomycin non-susceptibility, in nine daptomycin-non-susceptible *Staphylococcus aureus* clinical isolates (daptomycin MIC: >1 mg/L). Susceptibility to daptomycin, vancomycin, linezolid, oxacillin, telavancin and dalbavancin was studied. *walkR*, *agrA*, *cls1*, *cls2*, *fakA, pnpA*, *clpP*, *prs*, *rpoB*, *rpoC* and *mprF* were amplified by PCR and sequenced. The sequences were compared with the *S. aureus* ATCC 25923 complete genome (GenBank gi: 685631213) by using BLAST^®^ software. We did not find any changes in *walkR*, *pnpA*, *prs* and *clpP*. All isolates excepting isolate MSa5 showed a high number of significant mutations (between 13 and 25 amino acid changes) in *mprF*. Most isolates also showed mutations in the *rpo* genes, the *cls* genes and *fakA*. Daptomycin non-susceptibility in *S. aureus* clinical isolates seems to be reached through different mutation combinations when compared to *S. aureus* ATCC 25293. Especially *mprF* and *cls1* showed very high polymorphism in most isolates. Meanwhile, one isolate, MSa5, showed only single mutation in *mprF* (P314T).

## 1. Introduction

*Staphylococcus aureus* has shown a high capacity for developing resistance to each newer antistaphylococcal antibiotic developed. Antibiotic resistance in *S. aureus* may result in higher morbidity, mortality, lengths of hospitalization and health expenditures [1]. The cyclic anionic lipopeptide antibiotic daptomycin (DAP) [2] is becoming a main resource in therapy against multidrug-resistant *S. aureus*, especially methicillin-resistant *S. aureus* (MRSA), due to its activity against glycopeptide and linezolid-resistant MRSA [3,4]. The mechanism of action of DAP differs from conventional glycopeptides (vancomycin, teicoplanin) and is more like the cationic antimicrobial peptides produced by the immune system. Vancomycin and teicoplanin impair bacterial cell wall synthesis. They bind to the terminal dipeptide D-alanyl-D-alanine (D-Ala-D-Ala) within the disaccharide pentapeptide attached to lipid II and the forming PGN chain. This way, transglycosylation and transpeptidation are prevented, thus hindering proper wall synthesis [5,6]. In addition, vancomycin has other secondary actions, probably less important in the lysis of the microorganism, such as inhibition of RNA synthesis and alteration of wall permeability.

The action of DAP is based on binding to the bacterial cell membrane through its hydrophobic end in the presence of calcium ions [7,8,9]. This binding occurs both in the exponential growth phase and the stationary phase, causing membrane depolarization due to the loss of potassium ions from the cytoplasm [10]. This process leads to the disruption of multiple functions of the bacterial cell membrane, such as the synthesis of proteins, DNA and RNA without the need for the antimicrobial to penetrate the cytoplasm. Unlike other antimicrobials, the mechanism of action does not involve the lysis of bacterial cells [11] but rather bacterial cell death due to alterations in cellular homeostasis [7,12].

Intrinsic resistance to DAP by the hydrolytic cleavage of ester bonds inside the DAP molecule has been described, in absence of contact with this or other antibiotics,, among gram-positive bacteria with a low percentage of GC, such as staphylococci [13].

*Streptomyces* strains highly resistant to daptomycin (MIC ≥ 256 µg/mL) have been found to inactivate the antibiotic through two primary modes of daptomycin inactivation: ring hydrolysis, leading to linearization of the cyclic compound, and deacylation of the lipid tail [14]. Additionally, *Paenibacillus lautus* has been shown to inactivate daptomycin. The production of the inactivating activity in *P. lautus* is inducible by exposure to daptomycin, and some data suggest it might be caused by metalloesterases [14].

This “natural” resistance mechanism has been suggested to be associated with the natural production of DAP-like molecules by different microorganisms. Though this type of resistance has not been shown in clinically important bacteria yet, resistance genes coding for these DAP-inactivating enzymes might be a potential source of resistance if they could be captured by pathogenic bacteria, as has happened before for other resistance determinants, such as CTX-M enzymes, which originated in *Kluyvera* spp. and are now present in most clinically important enterobacteria [15].

Nevertheless, DAP non-susceptibility observed in isolates obtained both in vitro and from therapeutic failures in *S. aureus* seems to be mainly associated with electrostatic repulsion of the DAP–calcium complex from the cell surface due to an increase in the positive charge of the bacterial surface [16].

DAP-non-susceptible isolates obtained both in vitro and from therapeutic failures usually harbor mutations in genes associated with cytoplasmic membrane (*mprF*) and cell wall homeostasis (*yycFG*, also known as *walK*) and mutations in RNA polymerase subunits *rpoB*/*rpoC* [7,8]. DAP-non-susceptible isolates have been described, both in vitro and in vivo, lacking mutations in some of these genes but not in all of them [7,9,10,11]. Other authors have shown that DAP non-susceptibility may also involve the upregulation of genes associated with cell wall synthesis and turnover, such as the two-component regulator *vraSR* [12]. Otherwise, whole-genome-sequencing studies have shown that DAP-non-susceptible isolates may harbor mutations in other genes, such as *agrA*, *pnpA*, *pgsA*, *prs*, *cls2* and *clpP*, some of them associated with the cytoplasmic membrane and cell wall metabolism [10,11].

Therefore, the present study had the following aims: the identification of *S. aureus* strains (methicillin-resistant *Staphylococcus aureus* (MRSA) and methicillin-susceptible *S. aureus* (MSSA)) non-susceptible to DAP, isolated from three different hospitals in Spain; testing sensitivity to different drugs such as oxacillin, vancomycin, linezolid, dalbavancin and telavancin; and the amplification and sequencing of the main genes associated with DAP resistance.

## 2. Materials and Methods

### 2.1. Selection of Bacterial Strains

Nine *S. aureus* clinical isolates identified as DAP-non-susceptible *S. aureus* (Minimum Inhibitory Concentration, MIC > 1 mg/L) by microdilution conventional susceptibility systems (WalkAway, Perkin Elmer, Shelton, CT, USA) were included in this study. Seven of them had been isolated in hospitals in the province of Badajoz (Southwest of Spain) and two in the Hospital Universitario de Salamanca (Middle West of Spain). All isolates except MSa9 were from patients who had received daptomycin treatment, although data on duration of treatment and dosage were not accessible. To our knowledge, the patient from whom the MSa9 isolate was obtained did not receive treatment with daptomycin at any time.

Microorganisms were identified by MALDI-TOF mass spectrometry (MicroflexTM, Bruker Daltonics, Bremen, Germany). All the isolates used were preserved and stored at −80 °C in glycerol broth with 10% skimmed milk.

### 2.2. Antibiotic Susceptibility

Susceptibility to DAP, vancomycin (VAN), linezolid (LZD) and oxacillin (OXA) were tested by using a broth microdilution method (MicroScan WalkAway, Perkin Elmer, Waltham, MA, USA), according to manufacturer instructions. Telavancin (TLV) and dalbavancin (DLV) were studied by E-test (Liofilchem, Roseto degli Abruzzi, Italy). The microdilution sensitivity study was carried out using an automated commercial method (MicroScan WalkAway, Perkin Elmer, USA) in which antimicrobials are lyophilized so that a predetermined concentration is reached by adding a set amount of culture medium. The microorganism is inoculated in cation-adjusted Mueller Hinton broth supplemented with 2% NaCl for oxacillin sensitivity and 50 microg/mL calcium for DAP. An inoculum of the microorganism equivalent to 0.5 of the McFarland (McF) standard is inoculated and incubated at 37 °C for 20–24 h. The reading is carried out in an automated way in MicroScan equipment.

For the E-test study, a Mueller Hinton agar plate was inoculated with an inoculum equivalent to 0.5 McF; strips of the different antimicrobials were deposited on the inoculated agar, incubated at 37 °C for 24 h and read visually. For the DAP E-tests, it was not necessary to supplement the agar with Ca^2+^, as the DAP strips had it incorporated.

Methicillin resistance, in strains with E-test sensitivity to oxacillin, was verified by PCR specific to the *mecA* and *mecC* genes (the primers used appear in Table 1). The isolates were subjected to the E-test according to the manufacturer’s instructions. The susceptibility criteria for VAN, LZD, DLV, TLV and OXA were those proposed by the CLSI (VAN: <2 mg/L, susceptible; 4–8 mg/L, intermediate; >16 mg/L, resistant. LZD: <4 mg/L, susceptible; >8 mg/L, resistant. DLV: <0.25 mg/L, susceptible; intermediate and resistant categories not defined. TLV: <0.12 mg/L, susceptible; intermediate and resistant categories not defined. OXA: <0.5 mg/L, susceptible; >1 mg/L, resistant) [13]. The only categories considered for DAP were “susceptible” and “non-susceptible”, as CLSI reports only the sensitivity criterion (MIC < 1 mg/L).

### 2.3. DNA Extraction and Amplification of Genes Associated with DAP Non-Susceptibility in S. aureus

A suspension with turbidity equivalent to 1 McF was obtained from colonies grown for 24 h at 37 °C on blood agar. Then, 500 μL of the suspension was transferred to the automated nucleic acid extraction system NucliSENS^®^ easyMAG (bioMérieux, Marcy-l’Étoile, France). Nucleic acid extraction was performed according to manufacturer instructions. DNA was preserved at −80 °C. A series of genes previously associated with DAP non-susceptibility [7,14] was amplified and sequenced. Amplification was carried out in an Eppendorf Master Cycler^®^ thermal cycler (Eppendorf, Hamburg, Germany). All primers used in this study were designed specifically (Table 1) based on the *S. aureus* ATCC 25,923 complete genome sequence deposited in GenBank (gi: 685631213) using the Primer Blast^®^ application (NCBI, Bethesda, MA, USA). The primers were manufactured by Sigma-Genosys (The Woodlands, TX, USA), and the MasterMix^®^ PCR amplification mix (Promega, Madison, WI, USA), a premixed 2X solution of Taq DNA Polymerase, dNTPs and reaction buffer, was used for PCR. PCR conditions were as follows: 95 °C × 5 min (denaturation); 35 cycles of: 95 °C × 30 s, 1 min at the temperature reported in the supplementary section (Table S1) specific to each primer (hybridization), 72 °C × 1 min (elongation), and finally, 72 °C × 5 min (elongation).

### 2.4. Visualization of Amplicons and Purification of PCR Product

The products were subjected to electrophoresis in 2% agarose gel together with a size control (DNA Ladder 100 bp, Promega, Madison, WI, USA). Blue/Orange 6x Loading Dye (Promega, Madison, WI, USA) was used as a loading buffer, and RedSafe^®^ Nucleic Acid Staining Solution (Invitrogen, Carlsbad, CA, USA) was used as a fluorescent dye for DNA detection.

As a general rule, amplified DNA was cleaned for sequencing by using Exo-SAP It^®^ (Chang Biosciences, Tianjin, China). When DNA was purified from the gel, the MEGAquick-Spin Total Fragment DNA Purification kit (Intron Biotechnology, Gyeonggi-do, Republic of Korea) was used.

Amplicons were sequenced by the Sanger method in a 3500 Genetic Analyzer sequencer (Applied Biosystems, San Francisco, CA, USA) and visualized by using the software Chromas 2.5.1.0. Sequences were compared with the *S. aureus* ATCC 25,923 complete genome sequence deposited in GenBank (gi: 685631213). *S. aureus* ATCC 25,923 is commonly used as a control in standard laboratory tests. Comparison and analysis were carried out by using the BLAST^®^ software freely available on the NIH website.

## 3. Results

### 3.1. Antibiotic Susceptibility

DAP MICs ranged between 1 and 4 mg/L (Table 1). All the isolates were susceptible to VAN, LZD, TLV and DLV (Table 1). The MIC of DLV for the MSa3 isolate was higher (0.1 mg/L), though still in the susceptible range (break point: 0.25 mg/L). Three out of nine isolates (33.3%) were phenotypically MRSA (OXA MIC ≥ 4 mg/L) and four MSSA (44.44%) (OXA MIC < 2 mg/L). Two isolates (MSa4 and MSa7) were OS-MRSA (OXA MICs = 2 mg/L, PCR positive for *mecA*) (primers in Supplementary Section Table S1).

### 3.2. Mutation Profiles Associated with Non-Susceptibility

**Figure 1:**
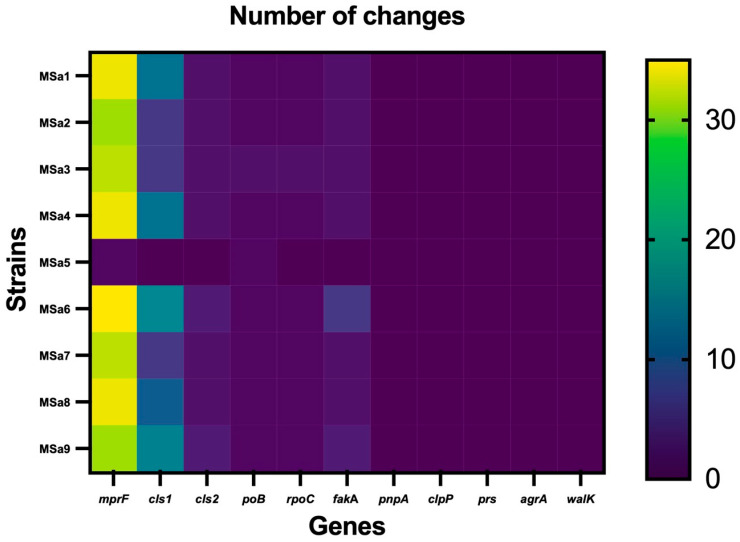
Number of mutations of each clinical isolate for each gene studied.

All the isolates, excepting MSa5, showed significant mutations in the genes *rpoB*, *mprF*, *rpoC*, *cls1*, *cls2* and *fakA*, while MSa5 showed significant mutations only in the *rpoB* and *mprF* genes (Figure 1) (Table 2, Table 3 and Table 4). We did not detect any significant mutations in the *walk*, *agrA*, *pnpA*, *clpP* and *prS* genes. The genes with the highest number of mutations were *mprF* (31–35 amino acid changes) and *cls1* (6–16 amino acid changes) (Figure 1).

The *mprF* gene presented a high number of mutations in all isolates (Table 2), excepting MSa5, which only had one mutation in this region: P314T. Mutations in this position also appeared in other clinical isolates, such as MSa1 (P314T) and MSa6 (P314L). Forty-three different mutations were identified in all in the *mprF* genes. Most isolates showed different combinations of these mutations. Most changes appeared in the central area of the genes, although we found changes throughout the genes. MSa6 had the highest number of mutations in *mprF*, exactly 35, followed by the strains MSa1, MSa4 and MSa8, with 34 changes.

As in *mprF*, we found a high number of changes in the genes that encoded for the cardiolipin synthase, especially in *cls1*. Only MSa5 showed no mutations in *cls1* (Table 3). Though a number of mutations appeared in more than one isolate, no isolates showed the same mutation profile. Only one mutation (I238V) appeared in all the isolates showing mutations in this gene. MSa6 was the isolate with the highest number of mutations in this gene (16 mutations). *cls2* only showed four different mutations (Table 3), half of which were shared by all the isolates, excepting MSa5, which did not show any mutation in this gene.

All the isolates, including MSa5, showed mutations in *rpoB*; however, all the isolates excepting MSa5 had mutations in *rpoC* (Table 4).

*fakA*, a protein also referred to as *mw1109* in previous studies, is a fatty acid kinase that has been associated with unsensitivity to DAP in mutants selected in vitro [10]. All the isolates tested in this study, excepting the clinical isolate MSa5, showed mutations in this gene (Table 4). Mutation profiles in *fakA* were more homogeneous than in *mprF* and *cls1*. Only MSa6 showed a mutation profile that was neatly different.

We did not identify changes in *walkR* in any of our nine clinical isolates. Other genes also associated occasionally with reduced susceptibility to DAP, such as *agrA, clpP, pnpA* and *prs*, did not show any significant mutations in this study.

In summary, we found two different mutation profiles. A group of eight isolates had changes in six genes (*mprF*, *rpoB*, *rpoC*, *cls1*, *cls2* and *fakA*), while one isolate had changes in only two genes (*mprF* and *rpoB*). *mprF* and *cls1* showed high numbers of mutations/isolate in most isolates, while for the other genes, the number of mutations/isolate was much shorter. In the absence of the Multilocus Sequence Typing (MLST) analysis, we could not rule out that the identified substitutions could potentially be associated with a specific genetic lineage rather than daptomycin resistance.

## 4. Discussion

DAP resistance seems to be based on complex mechanisms and has been associated with the presence of mutations in different genes, most of them involved in the metabolism and homeostasis of the cell membrane and, in some cases, of the bacterial wall [7,14,15]. A cause–effect relationship between specific mutations in specific genes and a specific increase in the MICs of DAP has hardly been described, and the repulsion hypothesis does not explain the resistance to DAP in all *S. aureus* isolates. Only some studies have shown that the replacement of the mutated *mprF* gene with a wild one reverses, at least partially, the MICs of DAP [16,18]. In the present study, the MIC interval of DAP ranged between 2 and 4 mg/L in all cases, except MSa2, which had an MIC of 1 mg/L. Although the non-sensitivity to daptomycin in *S. aureus* is MIC > 1 mg/L, due to its proximity to the range, MSa2 was not ruled out from this study.

Mutations in the *rpoB* and *mprF* genes have been reported in most staphylococcal clinical isolates showing DAP MIC increases [7,10,11]. *mprF* codes for the bifunctional enzyme MprF, which contributes to the positive charge of the cell membrane surface through the lysinylation of phosphatidylglycerol (PG) and the translocation of lysinylated PG (L-PG) from the inner to the outer leaflet of the cell membrane. The positively charged cell membrane surface resulting from this process helps repel the DAP molecule from the surface [19,20,21]. The association between *mprF* mutations and DAP non-susceptibility is supported by the finding that the inactivation of mutated *mprF* can reverse, at least partially, DAP non-susceptibility [22].

The mprF protein is composed of 14 transmembrane domains and a cytosolic C-terminal domain [11]. The first eight N-terminal transmembrane domains are involved in the translocation of L-PG to the outer CM. The next four transmembrane “central” domains are involved both in L-PG synthesis and flipping, while the cytosolic C-terminal domain is involved only in L-PG synthesis. Single-nucleotide polymorphism (SNP) in *mprF* genes has been reported in most staphylococcal clinical isolates showing DAP MIC increases [7,10,11] and is associated with gain of function. Nevertheless, some authors have shown that SNPs can also be found in 30% of DAP-susceptible isolates and that some DAP-susceptible isolates with MICs of 1 mg/L can show up to 30 amino acid changes in *mprF* [23], suggesting a high level of heterogeneity within *mprF* in some staphylococcal isolates. Nevertheless, the DAP-non-susceptible isolates studied by these authors [23] showed only one amino acid substitution in the mprF (L341S or L826F). Changes in these or in very near positions have been previously reported to be associated with DAP non-susceptibility (S829L, S295L, P314L, S337L, T345I, T345A, I420N) [7,8,11,17,24].

Unlike results reported by other authors, we found one SNP in *mprF* only in one isolate (MSa5). Mutations at the 314 position had been reported to be previously associated with DAP non-susceptibility, but as P314L [7] instead of P314T, as appears in MSa5. P314L was found in another strain (MSa6, Table 2). The other isolates had high numbers of mutations affecting the three areas. They had between six and eight mutations in the N-terminal “flippase” domains (mode: six mutations), seventeen and twenty mutations (mode: eighteen mutations) in the central, bifunctional domains and eight and nine mutations (mode: eight) in the phosphatidylglycerol lysinylation domain. Six isolates (MSa1, MSa4, MSa5, MSa6, MSa7 and MSa8) included in this wide group of mutations showed some amino acid changes in positions previously associated with DAP non-susceptibility (S295, P314, T345) [7,11,16], but other isolates (MSa2, MSa3 and MSa9) showed mutations previously not reported in association with DAP non-susceptibility. Moreover, they showed no mutations in the area comprising between S295 and I420, where most DAP non-susceptibility-associated mutations reported are located, though they showed more than 30 amino acid changes throughout the gene. This study reflects a large genetic variability between our clinical isolates and the strain used as a reference strain (*S. aureus* ATCC 25923), especially in some of the genes studied. Some of the mutations observed in the different genes have been previously described to be associated with daptomycin resistance in previous studies [7,8,11,16,17], but others have not. As already mentioned in the results section, the absence of MLST forced us to consider the possibility that at least some of these previously undescribed changes are not associated with daptomycin resistance but with the genetic variability existing between the different MLST types.

All the isolates showed *rpoB* mutations. The *rpoB* and *rpoC* genes encode for the bacterial RNA polymerase β and β’ subunits [7,8]. The mutations described in *rpoB* and *rpoC*, associated with DAP non-susceptibility, are different from those affected by other antibiotics, such as rifampicin [25]. Unlike *mprF* mutations that, at least in vitro, usually emerge at relatively early times during the selection process, *rpoB* and *rpoC* mutations emerge later [7]. Some studies have reported *rpoB* changes in mutants selected in vitro but not in clinical isolates [7], while other studies [11] did not detect *rpoB* changes in strains selected in vitro and detected them only in clinical isolates. Some authors [25,26] have shown that single-point mutations in *rpoB*, such as A477D or A621E, can reduce susceptibility to both daptomycin and vancomycin. Such mutations have been shown to cause cell wall thickening and reduction of the negative charge of the outer layer in *S. aureus*. All the isolates tested in this study showed a *rpoB* mutation (F737Y) that had not been previously described in non-susceptible isolates, whether clinical or obtained in vitro. Changes in the *rpoC* gene were also detected in all isolates, excepting MSa5. Single *rpoC* mutations (N341D) have also been associated with treatment failures in staphylococcal infections [27]. Mutations in the walkR sequence, a regulator of cell wall metabolism and virulence in *S. aureus, B. subtilis* and *S. pneumoniae* [28], have been associated with vancomycin resistance [29] and also with reduced DAP susceptibility, in association or not with *mprF* and *agrA* mutations [10,29].

*fakA* has only been associated with DAP non-susceptibility in one study, and only in mutants selected in vitro [10]. In that study, the authors found a L133H mutation associated with DAP non-susceptibility. In our study, this protein showed different changes in eight out of nine clinical isolates. Since the function of this protein is unknown, it is not possible to know its role in DAP susceptibility or even if there is really a cause–effect relationship.

*cls* genes encode for a cardiolipin synthase, and mutations in these genes have been associated with low DAP susceptibility in staphylococci [11]. Cls proteins are membrane-bound enzymes that synthesize cardiolipin, an important anionic membrane phospholipid, from the phosphatidyl moiety of two PG molecules [30]. While in logarithmic growth, PG is the main membrane phospholipid, in stationary growth conditions and under conditions of stress, such as unfavorable growth conditions or cell wall acting antibiotics, cardiolipin can accumulate up to 25–30% of the membrane phospholipid. *cls2* has been the most frequently reported *cls* gene in this aspect. In this study, all isolates excepting MSa5 showed mutations in this gene. Most isolates shared two mutations (V135I, H205R), and two of them added a third mutation (A459L, A471E). None of these mutations had been described previously. The mutations previously reported appeared between amino acids 20 and 60 (A23V, T33N, L52F, F60S) [11] in the two transmembrane domains described in this protein, while our mutations appeared outside these domains and the cardiolipin synthase active domains.

Mutations in *cls1* have been described with a much lower frequency in association to reduced DAP susceptibility. Nevertheless, we found mutations in this gene in the same isolates in which we found mutations in *cls2*, and the number of mutations in this gene/isolate ranged from six to sixteen. Most mutations found in the isolates tested in this study appeared outside the transmembrane and cardiolipin synthase active domains, but V18A (in *cls1*), which appeared in all isolates excepting MSa5 and G20A and appeared in MSa6 and MSa9 (Table 3), was in the first transmembrane domain, and I238V, which also appeared in all isolates excepting MSa5, and I421M (isolates MSa1, MSa4 and MSa9) appeared in the cardiolipin synthase domains. Results concerning *cls1* and *cls2* suggest that, though DAP non-susceptibility-associated mutations are more frequently described in *cls2*, in the isolates tested in this study, it seems more likely that the most transcendent mutations are those observed in *cls1*, since, unlike *cls2*, in almost all isolates, we found mutations that specifically affect the transmembrane and cardiolipin synthase functional domains. Mutations in these areas may have a summation effect with those found in *mprF*, since the negative charge provided by cardiolipin would be reduced or even suppressed, reducing the attraction between DAP and the bacterial membrane [31].

## 5. Conclusions

The present study confirms that the acquisition of daptomycin resistance may be associated with different mutational profiles. Therefore, various combinations of mutations in different genes may end up leading to the same result of low susceptibility to daptomycin. However, resistance is mainly associated with mutations in the *mprF* gene involved in the amount of and situation on the membrane of lysyl phosphatidyl glycerol molecules; *rpoB*, which encodes an RNA polymerase; and the *cls* genes, which encode a cardiolipin synthase involved in the metabolism of membrane phospholipids. In the isolates analyzed in this study, *mprF, cls1* and *cls2* showed much higher numbers of mutations than reported in other studies, many of which had not been described so far. There is no relationship between the number of changes in the genes studied and the degree of sensitivity (MICs). That is, we could find strains with the same MICs and different genetic profiles. Future studies should clarify the real role of each of these mutations.

## Figures and Tables

**Table 1:** Antibiotic susceptibility of the isolates studied (MIC, mg/L). DAP: daptomycin; VAN: vancomycin; OXA: oxacillin; LZD: linezolid; DLV: dalbavancin; TLV: telavancin. S: susceptible; R: resistant.

Strains	DAP	VAN	OXA	LZD	DLV	TLV
MSa1	4.0 (R)	1.0 (S)	16 (R)	2.0 (S)	0.064 (S)	0.125 (S)
MSa2	1.0 (S)	1.0 (S)	256 (R)	2.0 (S)	0.047 (S)	0.047 (S)
MSa3	4.0 (R)	2.0 (S)	0.125 (S)	2.0 (S)	0.100 (S)	0.125 (S)
MSa4 *	2.0 (R)	2.0 (S)	2.0 (S)	2.0 (S)	0.064 (S)	0.125 (S)
MSa5	4.0 (R)	1.0 (S)	0.064 (S)	2.0 (S)	0.047 (S)	0.125 (S)
MSa6	4.0 (R)	1.0 (S)	4.0 (R)	2.0 (S)	0.032 (S)	0.125 (S)
MSa7 *	2.0 (R)	1.0 (S)	2.0 (S)	2.0 (S)	0.064 (S)	0.094 (S)
MSa8	4.0 (R)	1.0 (S)	0.125(S)	2.0 (S)	0.047 (S)	0.125 (S)
MSa9	2.0 (R)	1.0 (S)	0.5 (S)	2.0 (S)	0.032 (S)	0.094 (S)

* MRSA (*mecA*+).

**Table 2:** Mutations in *mprF* gene.

N° of Isolate (MSax)	Amino Acid Mutations ^1^	Base Changes *
1, 2, 3, 4, 6, 7, 8, 9	V26A; N160D; A171V; L174F; Y194F; V223A; L371I; Y400F; I406L; I409T; L413F; V426A; A430V; I446V; L451I; I459L; I464V; F473L; V478I; K489R; I494L; V503I; A505M; N522K; E525D; D531N; D554N; N556T; I575L; T696E; N710E	T1353395C; A1353696G; C1353730T-T1353731A; G1353740T; A1353799T; T1353886C-C1353887T; C1354329A; A1354417T; A1354434T-C1354436A; T1354444C-T1354445A; A1354457C; T1354495C; C1354507T; A1354554G-T1354556A; C1354569A; A1354593T; A1354608G-C1354610T; T1354637A; G1354650A; A1354683C-A1354684G-A1354685C; A1354698C; G1354725A-T1354727A; G1354731A-C1354732T-A1354733G; T1354784A; G1354793T; G1354809A; G1354878A; A1354885C-T1354886A; A1354941T- C1354943A; A1355305G-C1355304A; A1355346G-T1355348A
1, 4	I375M; I461T	A1354343G; T1354600C-A1354601T
1, 5	P314T	C1354158A-G1354160T
6	I9V; G105A; P314L ^2^; P721T	A1353243G; G1353532C; C1354159T-G1354160T; C1355379
3	L291I	T1354089A
4, 7	T345I ^2,4,5,^	C1354252T
8	V287I; S295P ^3^; A500S	G1354077A-G1354079A; T1354101C; G1354716T- A1354718T

^1^ Amino acid numbering corresponds to their positions within the *mprF* gene. * Base numbering corresponds to the complete genome of *S. aureus* subsp. *aureus* strain ATCC 25923 (GenBank: CP009361.1). ^2^ Friedman [7]; ^3^ Friedman [7] and Peleg [11] S295L; ^4^ Mishra [8]; ^5^ Friedman [7], Mishra [8], Murthy [17] and Cameron [16]: T345A.

**Table 3:** Mutations in cardiolipin synthase genes (*cls1* and *cls2*).

Genes	N° of Strain (MSax)	Amino Acid Mutations ^1^	Base Changes *
*cls1*	1, 4, 6, 8, 9	K147Q; K170Q; H174K; N197K	A1306452C; A1306521C; C1306533A-T1306535A; T1306604A
	1, 4, 9	F389Y; I421M; N448K	T1307179A; C1307276G; T1307357G
	1, 2, 3, 4, 6, 7, 8, 9	I238V	A1306725G-T1306727A
	1, 2, 3, 4, 7, 8	E469K	G1307418A
	1, 2, 3, 4, 7, 8	Q2R; F3Y; S4T	A1306018G; T1306021A; T1306023A
	1, 2, 3, 4, 7, 8	V18A	T1306166C
	6, 9	V18A; G87A; V132I; A175V	T1306166C-C1306167A; G1306273C-A1306274G; G1306407A; C1306537T-T1306538G
	9	S43A; T44K	T1306140G; C1306144A-T1306145A
	6, 9	G20A	G1306072C-A1306073C
	6	V300E; G308K; P309S; L310F; S313A; V455A	T1306912A-T1306913A; G1306935A-G1306936A; C1306938T; G1306943C; T1306950G-A1306952G; T1307377C
	5	NC	NC
*cls2*	1, 2, 3, 4, 6, 7, 8, 9	V135I; H205R	G2125012A; A2125223G
	6	A471E	C2126021A
	9	I459L	A2125984T
	5	NC	NC

^1^ Amino acid numbering corresponds to their positions within the *cls1* and *cls2* genes. * Base numbering corresponds to the complete genome of *S. aureus* subsp. *aureus* strain ATCC 25923 (GenBank: CP009361.1). NC: no change.

**Table 4:** Mutations in *rpoB*, *rpoC* and *fakA* genes.

Genes	N° of Strain (MSax)	Amino Acid Mutations ^1^	Base Changes *
*rpoB*	1, 2, 3, 4, 5, 6, 7, 8, 9	F737Y	T545943A
	3	M513I	G545272A
*rpoC*	1, 2, 3, 4, 6, 7, 8, 9	V864I	G550011A-T550013A
	3	P100L	C547720T
	5	NC	NC
*fakA*	1, 2, 3, 4, 6, 7, 8, 9	L214I; D497E	C1191817A; T1192668A
	9	E431D	A1192470C
	6	I144V; E277K; Y287H; A513E	A1191607G; G1192006A; T1192036C; C1192715A
	5	NC	NC

^1^ Amino acid numbering corresponds to their positions within the *rpoB*, *rpoC* and *fakA* (*mw1109)* genes. * Base numbering corresponds to the complete genome of *S. aureus* subsp. *aureus* strain ATCC 25923 (GenBank: CP009361.1). NC: no change.

## Data Availability

The authors declare that data related to this research are available from the authors upon reasonable request.

## Supplementary Materials

The following supporting information can be downloaded at: https://www.mdpi.com/article/10.3390/pathogens13030206/s1, Table S1: Oligonucleotide specific primers.
