# Supplementary material for: Genomic Insights into Staphylococcus aureus Isolates Exhibiting Diminished Daptomycin Susceptibility
Source: Pathogens. 2024 Feb 26;13(3):206. doi: 10.3390/pathogens13030206 (PMC10974884; doi:10.3390/pathogens13030206)
Supplement: Supplementary file 1 [file pathogens-13-00206-s001.zip › pathogens-2866185-supplementary.pdf]

Supplementary section

Table S1. Oligonucleotide specific primers

Genes	Primer name	Primer sequence (5′ → 3′)	Annealing temp. (C degrees)
mecA	mecA-D	ggccaattccacattgtttc	60
	mecA-R	aaattgaacgttgcgatcaa	
mecC	mecC-D	aaaatcagagcgaggcaaaa	61
	mecC-R	tggctgaacccatttttgat	
walK	walK-D	ggtcgaaacgaatgaagtgg	62
	walK-R	tttggctgtcataggtgtcg	
agrA	agrA-D	ggtgaaggtcgtggtttagg	63
	agrA-R	gccagctatacagtgcatttg	
cls1	cls1.1-D	aggttctgaagaagctgcaaa	61
	cls1.1-R	ccaatgttaaatcctccgaca	
	cls1.2-D	tcggaggatttaacattggtg	61
	cls1.2-R	ttgaatcaaatgaaccacctctt	
cls2	cls2.1-D	tcgctctcacttgtccttca	64
	cls2.1-R	gtcacgtgtggcttgtgaat	
	cls2.2-D	gggcaaattggatatgttgg	60
	cls2.2-R	gaagcaaacatgcgagttca	
*fak*A	mw1109-1-D	ctttccaaaacgaaccatca	60
	mw1109-1-R	ctttccaaaacgaaccatca	
	mw1109-2-D	tgaagccaaagttgcaaaga	62
	mw1109-2-R	tgacattgttagcaactcgagaa	
pnpA	pnpA-D-1	actgataagggtgggacgat	63
	pnpA-R-1	ctggaccagtttcacctactga	
	pnpA-D-2	cacgtggtcaaactcaagca	61
	pnpA-R-2	cctaaatttgttacccgtcca	
clpP	clpP-D	cgtaaacgcataccaaccaa	62
	clpP-R	tgttgactccctcaacacga	
prs	prs-D	tcatgggttattgattggtga	60
	prs-R	ccaaagctgccatacttaaaaga	
mprF	mprF-1N-D	agataaccatattgttctgtttgaga	62
	mprF-1N-R	tgtttttatcgggcggtcta	
	mprF-2N-D	tcgtattccatgtttttgatgc	61
	mprF-2N-R	tctcattcgaattggacgttt	
	mprF-3N-D	ttgtcgcgacgttattcact	61
	mprF-3N-R	attttcattgcgcatcacac	
	mprF-4N-D	atatcataatttcggcaaccaa	60
	mprF-4N-R	ccagagcgttatgcagaaga	
rpoB	rpoB-1-D	agctaaaagcggatcaca	57
	rpoB-1-R	tgaggtgtgatagactca	
	rpoB-2-D	ggaaagagttgtacgtga	57
	rpoB-2-R	gccgtgaggtacacgta	
	rpoB-3-D	agcacgtgaagttagaga	57
	rpoB-3-R	tttgtgtttcaggagca	
	rpoB1.2-D	cagacgttgatgacgatgatg	62
	rpoB1.2-R	ccagttggacctggatctaca	

rpoC	rpoC-1N-D	aatgcaaatcaatcaaatagcacag	62
	rpoC-1N-R	tgttgcaaatcgtccaccatc	
	rpoC-2N-D	ctggtcaaagacttactcgtgc	62
	rpoC-2N-R	tgctaaatctgttgaatacttctgc	
	rpoC-3N-D	tacaactttaggtgaaggtggat	62
	rpoC-3N-R	tcaccgatagattgggctgc	
	rpoC-4N-D	acagatgctggtattgaacaaatg	62
	rpoC-4N-R	tcaacttctgcaactggtttagc	
